# The impact of intermediate-term alcohol abstinence on memory retrieval and suppression

**DOI:** 10.3389/fpsyg.2014.01396

**Published:** 2014-12-02

**Authors:** Viola L. Nemeth, Eszter Kurgyis, Gabor Csifcsak, Anikó Maraz, Denes A. Almasi, Gergely Drotos, Petronella Szikszay, Balint Ando, Zoltán Janka, Anita Must

**Affiliations:** ^1^Department of Psychiatry, Faculty of Medicine, University of SzegedSzeged, Hungary; ^2^Institute of Psychology, Faculty of Arts, University of SzegedSzeged, Hungary; ^3^Department of Clinical Psychology and Addiction, Institute of Psychology, Eötvös Loránd UniversityBudapest, Hungary; ^4^Faculty of Science and Informatics, University of SzegedSzeged, Hungary; ^5^Neuroimaging Research Group, Research Center for Natural Sciences, Hungarian Academy of SciencesBudapest, Hungary; ^6^Addiction Rehabilitation Center Based on the Minnesota Model, Hospital of SzigetvarSzigetvar, Hungary

**Keywords:** alcohol dependence, intermediate-term abstinence, relational memory, inhibition of retrieva, cognitive control

## Abstract

**Background:** The nature of episodic memory deficit in intermediate-term abstinence from alcohol in alcohol dependence (AD) is not yet clarified. Deficits in inhibitory control are commonly reported in substance use disorders. However, much less is known about cognitive control suppressing interference from memory. The Think/No-think (TNT) paradigm is a well established method to investigate inhibition of associative memory retrieval.

**Methods:** Thirty-six unmedicated patients with AD and 36 healthy controls (HCs) performed the TNT task. Thirty image–word pairs were trained up to a predefined accuracy level. Cued recall was examined in three conditions: Think (T) for items instructed to-be-remembered, No-think (NT) assessing the ability to suppress retrieval and Baseline (B) for general relational memory. Premorbid IQ, clinical variables and impulsivity measures were quantified.

**Results:** AD patients had a significantly increased demand for training. Baseline memory abilities and effect of practice on retrieval were not markedly different between the groups. We found a significant main effect of group (HC vs. AD) × condition (B, T, and NT) and a significant difference in mean NT–B scores for the two groups.

**Discussion:** AD and HC groups did not differ essentially in their baseline memory abilities. Also, the instruction to focus on retrieval improved episodic memory performance in both groups. Crucially, control participants were able to suppress relational words in the NT condition supporting the critical effect of cognitive control processes over inhibition of retrieval. In contrast to this, the ability of AD patients to suppress retrieval was found to be impaired.

## INTRODUCTION

Harmful effects of chronic alcohol consumption on the brain have long been a topic of major interest (Fitzhugh et al., 1960). Neuroanatomical alterations might well account for the impairment of various cognitive functions in alcohol dependence (AD; Chanraud et al., 2007). Previous research reported deficits in episodic memory in AD patients (Parker et al., 1974, 1976). Episodic memories require encoding, storage and retrieval of previously experienced events. These functions are primarily linked to structures of the medio-temporal lobe (Baddeley et al., 2009). The limbic system and particularly the hippocampus are highly susceptible to chronic excessive alcohol consumption (Oscar-Berman and Marinkovic, 2003; Beresford et al., 2006).

The episodic memory impairment in alcoholism is associated with reduction in the ability to learn complex novel information (Pitel et al., 2007a). A number of studies have demonstrated improvement of neuropsychological functioning in AD following a certain period of sustained abstinence (Kish et al., 1980; Claiborn and Greene, 1981; Fein et al., 1990). Findings suggest that significant improvement in most cognitive domains occurs over the first months to 1 year of abstinence (Munro et al., 2000; Sullivan et al., 2000; Fein et al., 2006). Pitel et al. (2007b) found episodic and working memory deficits that affected procedural learning strategies in very early abstinence. This deficit is presumed to be associated with hippocampus atrophy as a neuroanatomical correlate of the dysfunction (Pitel et al., 2007a). However, the nature of episodic memory problems in intermediate-term abstinence is not yet clarified.

Deficits in inhibitory control have previously been detected in substance use disorders (Goldstein and Volkow, 2002; Lubman et al., 2004). In AD the impairment in the ability to control impulses is still a debated question (Lipszyc and Schachar, 2010). A number of studies reported inhibitory control deficiencies in AD (Lawrence et al., 2009; Noel et al., 2013) whereas others found no significant deficit (Li et al., 2009; Schmaal et al., 2013). A potential explanation is yielded by the theoretical concept of inhibition. Oberauer defined two potentially distinct mechanisms relevant to inhibitory control: overcoming prepotent response and suppressing proactive interference from memory (Oberauer, 2009). Previous research focused predominantly on the control exerted over prepotent, but irrelevant or inappropriate responses. However, much less is known about relevant aspects of cognitive control suppressing interference from memory.

Recently, the notion was raised that the inconsistencies of findings regarding inhibitory control in AD might well be due to the heterogeneous character of the disorder as well as co-morbid factors such as impulsivity (Sjoerds et al., 2014). Impulsivity is considered both a determinant and a consequence of substance dependence (de Wit, 2009), whereas determinants of impulsivity have been linked consistently to inhibitory processes (Dalley et al., 2011).

The Think/No-think (TNT) paradigm has originally been designed to investigate inhibition of retrieval (Anderson and Green, 2001). However, the TNT task also involves learning of cue-target stimuli pairs thus activating associative memory processes. Stimuli pairs are studied up to a defined accuracy level to ensure proper encoding in the medial temporal lobe (MTL) including the hippocampus (Depue and Banich, 2012). After successfully building associative memory some pairings are trained further to improve subsequent retrieval, some are instructed to be intentionally forgotten, while the remaining items will serve as baseline memory. Reductions from baseline memory for “to be forgotten” associations suggest that cognitive control actually reduces accuracy and depletes memory processes (Depue et al., 2007; Depue, 2012).

The aim of the current study was to directly compare episodic memory performance and inhibition of retrieval in intermediate-term alcohol abstinence. To the best of our knowledge, this is the first study to employ the TNT paradigm in AD. The stage of intermediate-term abstinence would allow us to investigate the impact of chronic alcohol consumption on the ability to retrieve or suppress previously learned memory associations without the confounding effect of current alcohol use or symptoms of withdrawal. We aimed to measure inhibitory control over episodic retrieval in AD; to distinguish general memory impairments from episodic recollection and cognitive control of inhibition and to further assess effects of impulsivity on cognitive control over episodic memory.

## MATERIALS AND METHODS

### PARTICIPANTS AND PROCEDURES

Written informed consent was obtained from 72 participants (43 males and 29 females) including 36 patients (aged 21–61 years; *M* = 42.81, SD = 8.96) and 36 healthy controls (HCs; aged 24–63 years, *M* = 40.39, SD = 10.04). Patients were recruited from the inpatient addiction unit of the Hospital of Szigetvar, a unique and comprehensive healthcare provider for alcohol-dependent patients from all over the country. All patients were diagnosed with AD based on the DSM-IV criteria (APA, 2000). Alcohol dependent patients had an average abstinent period of 14.61 weeks (SD = 9.60). Patients were not taking any psychotropic medication at the time of participation. HC participants were required to have no history of AD or any other psychiatric disorder. The two groups were matched for age, gender and years of education. All participants with a significant neurological illness, a significant head injury, a history of mood disorder independent from alcohol use, a history of schizophrenia spectrum disorder and a history of drug dependence were excluded prior to the interview process. Patients presenting current withdrawal symptoms were also excluded. Exclusion criteria were identified based on medical records and chart notes of the treating physician specialized in psychiatry and addictology. Accurate inclusion criteria were further verified in the interview phase. Two patients were diagnosed with a major psychiatric disorder currently present and therefore excluded. Written informed consent was obtained from all participants after approval of the study protocol by the local Ethics Committee.

All participants were assessed using the Hungarian version of the National Adult Reading Test (NART) constructed to predict premorbid IQ measures (Nelson and Willison, 1991). All patients were requested to complete the Alcohol Use Disorders Identification Test (AUDIT) for demographic and alcohol consumption-related variables (Babor et al., 2001). The Beck Depression Inventory (BDI) was performed to quantify depressive symptoms (Beck et al., 1961). Additionally, the Derogatis’ Symptom Checklist-90 (SCL-90) was administered to evaluate clinical symptoms referring to psychological distress (Derogatis, 1977). The Delayed Discounting Test (Richards et al., 1999) and the Barratt Impulsiveness Scale were completed to objectively measure the level of impulsivity (Barratt, 1959; Patton et al., 1995). All interviews were conducted by two trained psychologists specialized in clinical psychology under the supervision of a board certified psychiatrist.

### EXPERIMENTAL PARADIGM

**FIGURE 1:**
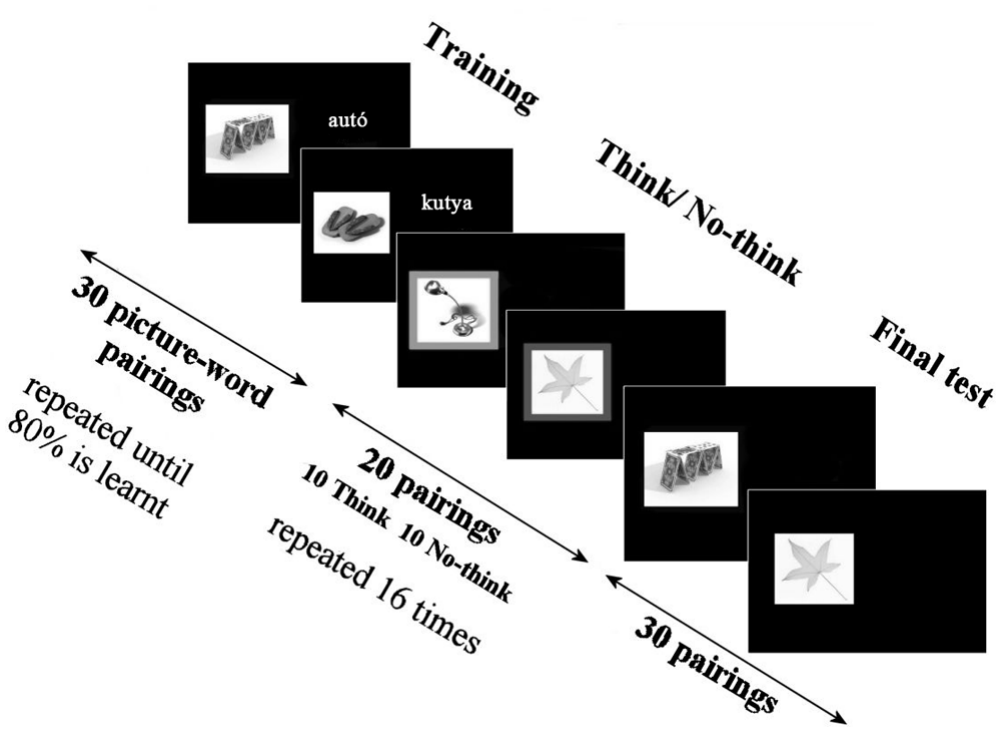
**The Think/No-think (TNT) paradigm involves the computerized presentation of 30 non-related, neutral picture–word pairs.** During each trial a full-colored image and a word were presented simultaneously for a period of 3.5 s. The experimental paradigm included three major phases. During the initial training block participants viewed the 30 image–word pairs. Training blocks consisting of the randomized presentation of all stimulus pairings were repeated until the participant succeeded to correctly identify at least 24 (i.e., 80% of the 30 pairs). Following completion of training all stimulus pairings were randomly classified into three groups (Baseline, Think, and No-think). Subsequently, the cue item was presented only and participants were instructed either to recall, i.e., “think” or suppress, i.e., “not think” of the target stimulus previously paired with the cue. Words previously studied with pictures now surrounded by a green colored frame, i.e., the T items, were asked to be recalled and named. In contrast to this, participants had to try to suppress the words which have been paired with images now presented with a red frame, these were the NT items. The final test consisted of the presentation of all 30 images originally studied.

A Hungarian version of the TNT task (Anderson and Green, 2001) was administered to assess episodic memory and inhibition of retrieval. The paradigm involves the computerized presentation of 30 non-related, neutral picture–word pairs (**Figure 1**). Stimuli were presented on a 17-inch color display controlled by a Windows-based computer using Presentation Software (version 16.5; Neurobehavioral Systems, Albany, CA, USA; http://www.neurobs.com/presentation). Picture–word pairs were constructed by random pairing, words were presented in Hungarian. During each trial a full-colored image and a word were presented simultaneously for a period of 3.5 s. The experimental paradigm included three major phases. The instructions were based entirely on test instructions of Anderson and colleagues applied in their original experiment (Anderson and Green, 2001). During the initial training block participants viewed the 30 image–word pairs. All participants were instructed to try and memorize the relational pairings for a recognition test to follow. Consecutively, participants were requested to name the associated item after the appearance of the cue with answers being recorded by the experimenter. Training blocks consisting of the randomized presentation of all stimulus pairings were repeated until the participant succeeded to correctly identify at least 24 (i.e., 80% of the 30 pairs) pairings. This accuracy level was set based on previous research practice. Participants were allowed to take breaks between training blocks if necessary. Immediately following completion of training all stimulus pairings were randomly classified into three groups: Baseline (B), Think (T), and No-think (NT). Grouping was rotated across subjects to ensure that each pair is assigned to each condition equally often. Subsequently, the cue item was presented only and participants were instructed either to recall, i.e., “think” or suppress, i.e., “not think” of the target stimulus previously paired with the cue. Words previously studied with pictures now surrounded by a green colored frame, i.e., the T items, were asked to be recalled and named. In contrast to this, participants had to try to suppress the words which have been paired with images now presented with a red frame, these were the NT items. Cue images were repeated eight times in a random order to allow testing of cognitive control over associative memory retrieval. The final test consisted of the presentation of all 30 images originally studied. Participants were requested to try to explicitly recall the word paired with the image during the initial training phase. Trials presented during the initial training phase only and not repeated subsequently serve as the B condition measuring baseline memory. Cued recall accuracy for T and NT pairs of stimuli was compared to memory for the B items (**Figure 1**).

### STATISTICAL ANALYSIS

All statistical analyses were performed using SPSS version 15.0 (IBM Corp., Chicago, IL, USA, 2006). To assess differences in demographic variables between the AD and control groups an analysis of variance (ANOVA) was carried out. Relationships between clinical measures and recalled items were examined using Pearson’s coefficient. Spearman’s Rho was determined for number of errors during the NART. Independent Samples Kruskal–Wallis Tests were performed to examine distribution of variables across groups. Group differences in TNT performance across the different conditions (T, NT, and B) were tested using the statistical approach of a general linear model, with condition as the within-subject factor and participant group as the between-subject factor (patient vs. control). Age was entered in the model as a covariate considering its possible confounding effect on episodic memory function and inhibition of retrieval (Anderson et al., 2011). Additionally two novel continuous variables were computed (T–B and NT–B) to assess group differences for the effect of practice and inhibition on retrieval. Group differences in number of block repetitions in the training phase serving to reach the predefined level were examined using Mann–Whitney U-probes. Statistical differences characterized by a *p* value below 0.05 were regarded as significant.

## RESULTS

### DEMOGRAPHIC MEASURES

Demographic characteristics of all participants are illustrated in **Table 1**. The *p* value exceeds 0.05 for all measures supporting that the two groups are comparable in age, years of education and NART errors. Chi-square tests showed no significant differences in gender (Chi-square = 0.921, *df* = 1; **Table 1**).

**Table 1:** Demographic characteristics of the alcohol dependence (AD) and healthy control (HC) group.

	HC mean (SD) *N* = 36	AD mean (SD) *N* = 36	Statistics^a^
Gender (male/female)	21/15	22/14	*F*(1,70) = 0.056, *p* = 0.813
Age	40.39 (10.04)	42.81 (8.96)	*F*(1,70) = 1.162, *p* = 0.285
Education (years)	13.10 (2.84)	12.67 (2.66)	*F*(1,70) = 0.654, *p* = 0.421
NART errors	8.77 (7.81)	12.083 (10.50)	*F*(1,70) = 2.263, *p* = 0.137

*NART, National Adult Reading Test. ^a^Analysis of variance (ANOVA)*.

### TNT PERFORMANCE

**FIGURE 2:**
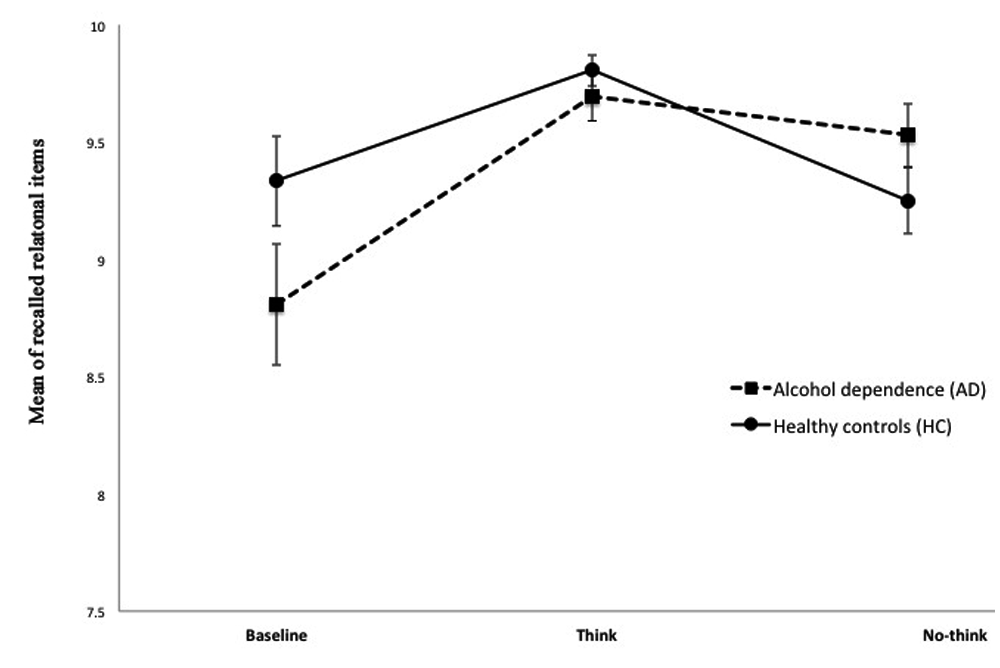
**Means of correctly recalled trials across TNT conditions are presented.** We found a significantly different pattern of performance for AD and HC when assessing the effect of inhibition on retrieval. At the same time the two groups did not differ significantly in their baseline memory ability as well as effect of practice on retrieval.

The means of correctly recalled trials across TNT conditions is presented in **Figure 2**. We found a significant main effect of group (HC vs. AD) × condition (B, T, and NT) [Greenhouse–Geisser *F*(1.568,108.198) = 5.408, *p* ≤ 0.01, observed power 0.767] after correcting for the potentially confounding effect of age. There were no significant group differences between AD and HC when comparing each testing condition separately: Baseline *F*(1,70) = 2.707, *p* = 0.104, Think *F*(1,70) = 0.807, *p* = 0.372, NT *F*(1,70) = 2.037, *p* = 0.156. In addition to this we compared the two newly computed variables between the two groups and found a significant difference for NT–B [*F*(1,70) = 6.400, *p* ≤ 0.01]. In contrast to this T–B was not significantly different between the two groups [*F*(1,70) = 1.521, *p* = 0.222]. We believe that these results support the idea that there is a significantly different pattern of performance for AD and HC when assessing the effect of inhibition on retrieval. While the two groups did not differ essentially in their baseline memory ability as well as effect of practice on retrieval, the ability to inhibit memory retrieval seems altered in the AD group. The mean NT–B score for HC group was negative, reflecting the effect of the instruction to suppress the retrieval of NT items. As opposed to this, mean NT–B scores for AD patients remained positive, indicating that the instruction to inhibit retrieval did not lead to a significant decline in episodic recall.

We found a significant difference between the AD and control groups when comparing the number of block repetitions during the training phase. AD patients had a significantly increased demand for training in order to reach the same levels of accuracy as the control group [*U*(72) = 518.15, *p* ≤ 0.05].

### CORRELATIONS WITHIN THE AD GROUP

The descriptive statistics of self-report measures registered in the patient group is presented in **Table 2**. Several clinical symptom measures correlated with performance achieved on the TNT task (see **Table 3**). Measures of Delayed Discounting Task (DDT) correlated negatively with the number of correctly recalled words in the training phase [DDT: *R*(36) = -0.405, *p* ≤ 0.05]. We found a negative correlation between depression and anxiety symptom severity, as well as level of symptomatic distress and the NT score [BDI: *R*(36) = -0.343, *p* ≤ 0.05, SCL-90 depression index: *R*(36) = -0.465, *p* ≤ 0.01, anxiety index: *R*(36) = -0.438, *p* ≤ 0.01, SCL-90 positive symptom distress index: *R*(36) = -0.445, *p* ≤ 0.01].

**Table 2:** Clinical measures of the alcohol-dependent group.

	Minimum	Maximum	Mean	Standard deviation
AUDIT total score	19.00	40.00	31.10	6.12
BDI total score	1.00	28.00	10.98	7.10
BIS total score	50.00	94.00	68.54	9.61
DDT	0	0.94	0.08	0.17
SCL-90 somatization index	0	2.33	0.76	0.64
SCL-90 compulsive index	0.11	3.22	1.29	0.73
SCL-90 interpersonal sensitivity index	0	3.22	1.06	0.87
SCL-90 depression index	0.08	3.54	1.35	0.99
SCL-90 anxiety index	0	3.10	1.05	0.77
SCL-90 hostility index	0	2.17	0.65	0.60
SCL-9 phobic anxiety index	0	1.71	0.68	0.52
SCL-90 paranoid ideation index	0	3.17	0.92	0.76
SCL-90 psychoticism index	0	2.00	0.71	0.58
SCL-90 global severity index	0	0.20	0.09	0.06
SCL-90 positive symptom total	4.00	76.00	45.92	20.11
SCL-90 positive symptom distress total	1.00	3.12	1.80	0.60

*AUDIT, Alcohol Use Disorders Identification Test; BDI, Beck Depression Inventory; BIS, Barratt Impulsiveness Scale; DDT, Delayed Discounting Task; SCL-90, Symptom Checklist-90*.

**Table 3:** Correlations between clinical measures and performance on the Think/No-think task in the AD group.

	Number of repetitions in learning phase	Number of recalled words				
	
		Training phase	Final test	Baseline	Think	No-think
**Pearson coefficient (*R*)**
DDT	0.311	-0.405*	-0.206	-0.313	0.221	-0.020
BIS total score	-0.196	-**0.335**	-0.078	-0.098	-0.043	-0.002
SCL-90 compulsive index	0.040	-0.125	-0.197	-0.082	-0.055	-0.359*
SCL-90 interpersonal sensitivity index	0.159	-0.415*	-0.377*	-0.334	-0.097	-0.356*
SCL-90 depression index	0.134	-0.312	-0.350*	-0.269	-0.015	-0.465**
SCL-90 anxiety index	0.231	-0.346*	-0.393*	-0.361*	0.019	-0.438**
SCL-90 hostility index	0.057	-0.350*	-0.074	-0.082	0.065	-0.101
SCL-90 phobic index	-0.033	-0.459**	-0.213	-0.299	-0.031	-0.010
SCL-90 paranoid ideation index	0.183	-0.388*	-0.269	-0.283	0.076	-0.282
SCL-90 global severity index	0.107	-0.411*	-0.339*	-0.288	-0.040	-0.380*
SCL-90 positive symptom distress total	0.115	-0.377*	-0.327	-0.256	0.010	-0.445**

*Correlation is significant at the 0.05 level; **Correlation is significant at the 0.01 level.

*DDT, Delayed Discounting Task; BIS, Barratt Impulsiveness Scale; SCL-90, Symptom Checklist-90*.

The relationship of NART errors and final TNT test performance revealed a significant negative correlation [*R*(36) = -0.407, *p* ≤ 0.05]. Final TNT test performance was assessed for all 30 relational items at the end of the examination.

## DISCUSSION

The results of this study provide novel and compelling evidence for the impact of intermediate-term alcohol abstinence on memory retrieval and suppression. To the best of our knowledge, this was the first study to employ the TNT paradigm in AD to directly compare episodic memory performance and inhibition of retrieval. Examining AD patients in intermediate-term abstinence allowed us to investigate the impact of chronic alcohol consumption on the ability to retrieve or suppress previously learned memory associations without the confounding effect of current alcohol use or symptoms of withdrawal. Our current results found no significant difference in baseline memory abilities between the two groups. However, it has to be noted that AD patients had a significantly increased demand for training in order to reach the same levels of accuracy as the control group. In addition to this, the relation between NART measures and final relational memory test performance might indicate the importance of premorbid IQ for more general episodic memory abilities. The instruction to focus on retrieval improved episodic memory performance in both groups with no essential difference. Crucially, the instruction to try and suppress retrieval of NT items resulted in a significantly different pattern for the AD and HC group. Control participants were able to suppress relational words in the NT condition supporting the critical effect of cognitive control processes over inhibition of retrieval. While the pattern of results was statistically comparable across groups for the B and T conditions, it reversed for the critical NT items. In this condition the ability of AD patients to suppress retrieval was found to be impaired.

There is evidence for the episodic memory performance to normalize over an ∼6-month period of sustained abstinence (Munro et al., 2000; Fein et al., 2006). As opposed to that AD patients who relapsed showed more severely impaired memory related cognitive performance not accounted for by a general executive dysfunction (Pitel et al., 2009). In addition to this, Pitel et al. (2010) found AD patients to present only mild to moderate deficits of explicit memory capacities. Our current findings are in accordance with the notion that episodic memory deficits might either be mild or even improve in the course of abstinence in AD. According to our results the baseline relational memory performance of AD patients was not significantly worse compared to the control group. Strikingly, repeated training on retrieval and the instruction to try and remember certain items had a beneficial effect on TNT performance in the T condition.

Reductions from baseline in the NT condition suggest that cognitive control exerted over inhibition of retrieval actually reduces accuracy and depletes memory processes. This is supported by our results derived from the HC group. In contrast to this, NT scores for the AD group increased. Intermediate-term abstinent patients with AD have been reported to show marked dysfunctions in the generalization of associations (Mattyassy et al., 2012). This impairment might be indicative of diminished episodic memory performance and relate to dysfunction of MTL structures. Functionally relevant microstructural changes in brain regions serving episodic memory functions have been reported in AD patients (Chanraud et al., 2009). Neuroanatomical correlates of relational encoding, as well as cognitive control over associative memory processes and inhibition of retrieval have been assessed taking advantages of a combination of the behavioral approach and neuroimaging methods. Results derived from functional magnetic resonance imaging (fMRI) as well as event related potential (ERP) electroencephalography (EEG) studies support the role of interaction between the lateral prefrontal cortex (LPFC) including the middle frontal gyrus (MFG) and MTL structures involving the hippocampus (Bergstrom et al., 2007; Waldhauser et al., 2012; Detre et al., 2013). Findings from fMRI studies signify increased activation of the MFG and adjacent areas for the NT condition whereas the hippocampus showed decreased overall activity during cued recall testing. Strikingly, actually forgotten items of the NT condition elicited an increase in hippocampal activation as compared to all other trials but solely in the first part of the experiment. Conversely, the initial increase was followed by the largest deplete in activation during the final phase. In the light of this pattern a potential explanation might be that inhibition of retrieval derived from the MFG induces a complex mechanism beginning with the association of inhibitory processes to the to-be-forgotten NT stimuli. Comprehensive analysis of findings from neuroimaging studies support the role of MFG on the inhibitory modulation of the hippocampus suggesting that successful cognitive control over memory retrieval and cued recall is associated with an inhibitory effect of the MFG on the hippocampus (Depue, 2012). While the vast majority of the TNT literature supports the notion that intentional cognitive control improves inhibition of retrieval, it has to be stated that some studies did not replicate these results (Mecklinger et al., 2009; Dieler et al., 2010). However, participant instructions (Racsmány et al., 2012), demand on cognitive processes and especially working memory as well as strategy might significantly influence behavioral results on the TNT task, which need further exploration (Raaijmakers and Jakab, 2012; Festini and Reuter-Lorenz, 2013).

In AD the prefrontal cortex (PFC) and its subterritories seem particularly vulnerable to chronic ethanol consumption (for a review see Moselhy et al., 2001). Findings also indicate that disruption in PFC function plays a crucial role in recovery difficulties and increased relapse risk (Seo et al., 2013). Recent evidence derived from animal studies shows that abstinence from alcohol in rats with a history of significant alcohol intake produced dysregulation of the medial PFC resulting in an impairment of executive control processes. Notably, the deficit typically occurred during acute (first days of abstinence) but not protracted (16–68 days) abstinence suggesting the potential for improvement with the length of abstinence (George et al., 2012).

Aspects of impulsivity have frequently been linked to the mechanism of inhibitory control (de Wit, 2009; Dalley et al., 2011). A number of studies provide support for the impairment of inhibitory processes in AD (Li et al., 2009; Noel et al., 2001). Above this, measures of inhibitory control were proposed as a predictor of problem drinking in adolescents at risk for AD (Nigg et al., 2006). Here we found higher levels of impulsivity to be associated with impaired relational encoding in the AD group.

The close relationship between AD and mood disorders has been studied extensively and firmly established (Helzer and Pryzbeck, 1988; Ross et al., 1988; Regier et al., 1990; Grant and Harford, 1995; Kessler et al., 1997). Findings report a high co-morbidity between AD and depression or anxiety (de Graaf et al., 2002; Boschloo et al., 2011). Notably, remitted or current AD represents a significantly increased risk for chronically persisting depressive and/or anxiety disorders (Boschloo et al., 2012). Trait anxiety present after 3 weeks of abstinence was found to represent a great relapse risk (Driessen et al., 2001). While the TNT literature consistently indicates that patients diagnosed with depression have difficulties with memory inhibition (Cottencin et al., 2008; Hertel and Mahan, 2008) it is unclear whether AD patients with co-morbid depression and anxiety resemble patients suffering from anxio-depressive disorders alone. Sjoerds et al. (2014) found AD patients not to be more impulsive than patients with depression and anxiety symptoms solely, but they did reveal inhibition impairments in the AD group which correlated with increased disorder severity. The current findings support the idea of a relevant effect of anxio-depressive symptoms on inhibitory control in intermediate-term alcohol abstinence. However, different mechanisms might be involved and alternative aspects might have to be considered for depression and/or anxiety in AD as compared to anxiety and depression alone.

In summary, our current study provides novel evidence for a deficit to exert inhibition of retrieval by applying the TNT paradigm in AD. Relational encoding was significantly different between the two groups with an increased demand for training in AD. However, associative recall ability in intermediate-term abstinence was not found to be significantly impaired when compared to HCs. Crucially, the instruction to try and suppress retrieval did not reach the level of the HC group for AD patients in intermediate-term abstinence. A number of questions concerning the exact nature and underlying neuronal correlates of inhibitory control processes in AD and along the process of abstinence still remain. However, by a thorough exploration of how current clinical signs affect executive cognitive control processes in the daily life of AD patients, caregivers might be able to target more specific therapeutic interventions. Above this, the ability to exert control over intrusive memories of potentially appealing cues might be of crucial importance in the long-term process of sustained abstinence.

## Conflict of Interest Statement

The authors declare that the research was conducted in the absence of any commercial or financial relationships that could be construed as a potential conflict of interest.
